# Supplementary material for: Implications of OPRM1 and CYP2B6 variants on treatment outcomes in methadone-maintained patients in Ontario: Exploring sex differences
Source: PLoS One. 2021 Dec 15;16(12):e0261201. doi: 10.1371/journal.pone.0261201 (PMC8673616; doi:10.1371/journal.pone.0261201)
Supplement: S2 File — (DOCX) [file pone.0261201.s002.docx]

*Implications of OPRM1 and CYP2B6 Variants on treatment outcomes in MMT patients in Ontario: Exploring sex differences*

S2 FILE

**Data clean-up and quality control**The following clean-up and quality control steps were conducted on the Pilot GENOA and GENOA data, which were merged into and analyzed as one dataset[1,2]. All analyses were performed on PLINK 1.90 and the RStudio interface of R i386 3.5.1[3–5].

First, the genotyped files were converted into .bed, .bim, and .fam files and merged into one dataset. Chromosomes that were marked as “bad” through genotyping were removed.

All samples that were genotyped were cross-referenced with the sample shipment documents to ensure that there were no missing samples.

Missingness per sample and per SNP was estimated using PLINK’s --missing flag. Samples and SNPs with more than 10% (at early QC stages), and 5%, (later in the QC process) were removed.

Four sets of samples were genotyped twice across the Pilot and GENOA data. For each duo of duplicates, the samples with the lowest missingness rates were kept.

Samples with discordant sex information were identified using the --check-sex flag in PLINK. Chromosome X’s inbreeding coefficient was graphed for males and females separately. Males with a coefficient ≥ 0.8 were kept; females with a coefficient ≤ 0.4 were kept.

The sample heterozygosity rates were checked. The resultant values of the heterozygosity rate were calculated using the equation “(N(NM)-O(Hom))/N(NM)”. A histogram was graphed of the heterozygosity rate, and the threshold was determined to be 0.22. Samples with a calculated rate of less than or equal to 0.17 were checked to be of Native American ethnicity. One sample was removed.

Heterozygous haploids and nonmale Y chromosome genotype calls were set as missing.

A principal component analysis was conducted as part of the ethnicity checks. The self-reported ethnicities of the samples were plotted against the genetically determined ethnicities (through principal component vectors) to visually highlight any outliers. Outliers were defined as samples whose genetically determined ethnicities fall too far from the self-reported ethnicities. Samples whose ethnicities were corrected were those that were determined to possibly partially belong to the genetically determined ethnic group (ex. self-reported as 'European' but is 'mixed European and Native North American'). Samples that failed the ethnicity check were removed.

Samples with high relatedness values (PLINK’s --genome output PI_HAT>=0.2) were identified. Along with samples that were believed to be duplicates, have failed the sex check, ethnicity check, and/or genotyping, they were visualized on their respective plate positions to see if any unusual patterns could be observed. Any newly identified duplicates were checked against the case report forms to verify their duplicate status. All verified duplicates were then removed.

A threshold of p<1E-6 was used to remove SNPs that significantly deviated from Hardy-Weinberg equilibrium.

**Pre-imputation and imputation**

To prepare the data for imputation by the Michigan Imputation Server[6], the following steps were run on a Linux operating system.

The build was updated using resources and instructions outlined on Will Rayner’s site (<https://www.well.ox.ac.uk/~wrayner/strand/>)[7].

The reference alleles for the European sample subset were set up to match those from HRC reference panel, and those of other ethnicities to match 1000 Genomes reference panel. The frequency file used for the 1000 Genomes match was taken from the McCarthy Group tools (<https://www.well.ox.ac.uk/~wrayner/tools/>)[8]. Non-European ethnicity subsets were matched to those of 1000 Genomes, as labelled on the Mathgen site (<https://mathgen.stats.ox.ac.uk/impute/1000GP_Phase3.html>)[9]. SNPs with high MAF (MAF>0.4) and those that don't match the respective reference panel (HRC or 1000G) were removed.

Since at that stage only the European subset had a sample size large enough for the purpose of our analysis (other ethnicities of less than 100 samples would not be powered enough for ethnically-stratified analysis), only samples of European descent were submitted for imputation and later analyzed.

With regards to SNPs of interest for this study, SNPs rs73568641, rs7451325, rs2279343, and rs10403955 were imputed.

Phasing was done using Eagle2 and imputation using Minimac4, with the HRC reference panel[10,11].

**Post-imputation filtering and quality control**

The following steps were performed using a virtual machine instance and cloud storage supported by the Google Cloud Platform (<https://console.cloud.google.com/>)[12]. Imputed individual chromosome files were recoded from .vcf to .ped/.map files, and then to .bed/.bim/.fam files before being merged into one file on PLINK for easy handling.

The Rsq values were used for filtering. SNPs with equal to or less than 0.3 rsq were identified to be of low quality and removed. Further, SNPs with MAF<0.05 were removed.

**References**

1. Samaan Z, Bawor M, Dennis B, Plater C, Varenbut M, Daiter J, et al. Genetic influence on methadone treatment outcomes in patients undergoing methadone maintenance treatment for opioid addiction: a pilot study. Neuropsychiatr Dis Treat [Internet]. 2014 Aug;1503. Available from: http://www.dovepress.com/genetic-influence-on-methadone-treatment-outcomes-in-patients-undergoi-peer-reviewed-article-NDT

2. Anderson CA, Pettersson FH, Clarke GM, Cardon LR, Morris AP, Zondervan KT. Data quality control in genetic case-control association studies. Nat Protoc [Internet]. 2010 Sep 26;5(9):1564–73. Available from: http://www.nature.com/articles/nprot.2010.116

3. Purcell S, Neale B, Todd-Brown K, Thomas L, Ferreira MAR, Bender D, et al. PLINK: A Tool Set for Whole-Genome Association and Population-Based Linkage Analyses. Am J Hum Genet [Internet]. 2007 Sep;81(3):559–75. Available from: https://linkinghub.elsevier.com/retrieve/pii/S0002929707613524

4. Cahn ML, Conner MM, Schmitz OJ, Stephenson TR, Wehausen JD, Johnson HE. Disease, population viability, and recovery of endangered Sierra Nevada bighorn sheep. J Wildl Manage [Internet]. 2011 Nov;75(8):1753–66. Available from: http://doi.wiley.com/10.1002/jwmg.232

5. R Core Team. R: A language and environment for statistical computing [Internet]. Vienna, Austria: R Foundation for Statistical Computing; 2020. Available from: https://www.r-project.org/

6. Das S, Forer L, Schönherr S, Sidore C, Locke AE, Kwong A, et al. Next-generation genotype imputation service and methods. Nat Genet [Internet]. 2016 Oct 29;48(10):1284–7. Available from: http://www.nature.com/articles/ng.3656

7. Rayner W. Strand Home [Internet]. [cited 2020 Aug 21]. Available from: https://www.well.ox.ac.uk/~wrayner/strand/

8. Rayner W. McCarthy Tools [Internet]. [cited 2020 Aug 21]. Available from: https://www.well.ox.ac.uk/~wrayner/tools/

9. 1,000 Genomes haplotypes -- Phase 3 integrated variant set release in NCBI build 37 (hg19) coordinates [Internet]. 2015 [cited 2020 Aug 21]. Available from: https://mathgen.stats.ox.ac.uk/impute/1000GP_Phase3.html

10. Loh PR, Danecek P, Palamara PF, Fuchsberger C, Reshef YA, Finucane HK, et al. Reference-based phasing using the Haplotype Reference Consortium panel. Nat Genet [Internet]. 2016 Nov 1 [cited 2020 Aug 21];48(11):1443–8. Available from: /pmc/articles/PMC5096458/?report=abstract

11. Das S. Next Generation of Genotype Imputation Methods [Internet]. University of Michigan; 2017 [cited 2020 Aug 21]. Available from: https://deepblue.lib.umich.edu/bitstream/handle/2027.42/138466/sayantan_1.pdf?sequence=1

12. Google Cloud. Google Cloud [Internet]. 2020 [cited 2020 Aug 21]. Available from: https://cloud.google.com/

**
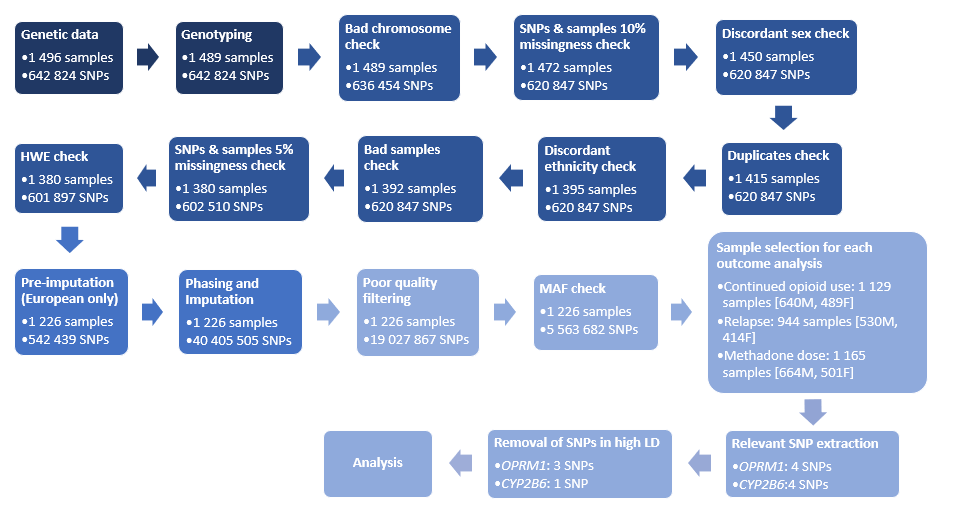
**

**Figure A. Flowchart of sample and SNP count changes throughout genotyping, quality control checks, and imputation.** HWE refers to Hardy-Weinberg equilibrium, MAF to minor allele frequency, M to the male sample, F to the female sample, and LD to linkage disequilibrium.

| 1. ***OPRM1***   **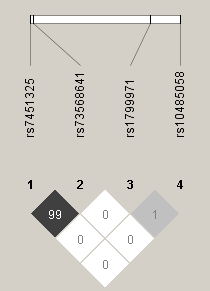** | 1. ***CYP2B6***   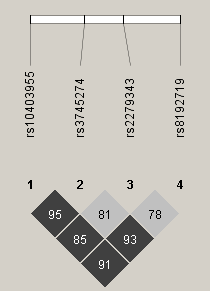 |
| --- | --- |

**Figure B. Linkage disequilibrium plots.** HaploView was used to visualize SNPs in linkage disequilibrium and calculate r-squared coefficients. The r-squared coefficient values (x100) are represented inside the plot.

**Association Tables**

For all the tables below: *P<0.05, **P<0.1. ADD is the additive regression test after adjustment for the covariates listed below and 5 principal component vectors. A1 represents the minor and tested (reference) allele. OR is odds ratio and BETA is the beta coefficient for the regression. STAT is the T-statistic and P is the p-value for it. The effect of the principal components is not displayed in the tables below.

| **Table A. Continued opioid use – *OPRM1*** | | | | | | |
| --- | --- | --- | --- | --- | --- | --- |
| **SNP** | **N** | **A1** | **TEST** | **OR** | **STAT** | **P** |
| **rs73568641** | 1129 | C | ADD | 0.84 | -1.27 | 0.20 |
|  |  |  | Age | 0.99 | -2.04 | 0.04 |
|  |  |  | Gender | 0.83 | -1.24 | 0.21 |
|  |  |  | Dose | 1.00 | -1.21 | 0.23 |
|  |  |  | Duration on MMT | 0.99 | -3.67 | 2.41E-4 |
| *Male* | 640 |  | ADD | 0.99 | -0.07 | 0.95 |
|  |  |  | Age | 0.98 | -2.22 | 0.03 |
|  |  |  | Dose | 1.00 | -1.72 | 0.09 |
|  |  |  | Duration on MMT | 0.99 | -2.65 | 8.13E-3 |
| *Female* | 489 |  | ADD | 0.71 | -1.66 | 0.1** |
|  |  |  | Age | 0.99 | -0.63 | 0.53 |
|  |  |  | Dose | 1.00 | 0.32 | 0.75 |
|  |  |  | Duration on MMT | 0.99 | -2.64 | 8.34E-3 |
| **rs1799971** | 1129 | G | ADD | 0.97 | -0.16 | 0.88 |
|  |  |  | Age | 0.99 | -2.03 | 0.04 |
|  |  |  | Gender | 0.83 | -1.19 | 0.23 |
|  |  |  | Dose | 1.00 | -1.14 | 0.26 |
|  |  |  | Duration on MMT | 0.99 | -3.65 | 2.58E-4 |
| *Male* | 640 |  | ADD | 1.11 | 0.47 | 0.64 |
|  |  |  | Age | 0.98 | -2.27 | 0.02 |
|  |  |  | Dose | 0.99 | -1.74 | 0.08 |
|  |  |  | Duration on MMT | 0.99 | -2.65 | 8.08E-3 |
| *Female* | 489 |  | ADD | 0.87 | -0.51 | 0.61 |
|  |  |  | Age | 0.99 | -0.58 | 0.57 |
|  |  |  | Dose | 1.00 | 0.45 | 0.66 |
|  |  |  | Duration on MMT | 0.99 | -2.60 | 9.23E-3 |
| **rs10485058** | 1129 | G | ADD | 0.96 | -0.28 | 0.78 |
|  |  |  | Age | 0.99 | -2.05 | 0.04 |
|  |  |  | Gender | 0.84 | -1.17 | 0.24 |
|  |  |  | Dose | 1.00 | -1.14 | 0.25 |
|  |  |  | Duration on MMT | 0.99 | -3.63 | 2.85E-4 |
| *Male* | 640 |  | ADD | 0.89 | -0.52 | 0.60 |
|  |  |  | Age | 0.98 | -2.24 | 0.03 |
|  |  |  | Dose | 1.00 | -1.73 | 0.08 |
|  |  |  | Duration on MMT | 0.99 | -2.64 | 8.22E-3 |
| *Female* | 489 |  | ADD | 1.00 | 0.01 | 0.99 |
|  |  |  | Age | 0.99 | -0.57 | 0.57 |
|  |  |  | Dose | 1.00 | 0.48 | 0.63 |
|  |  |  | Duration on MMT | 0.99 | -2.59 | 9.51E-3 |

| **Table B. Continued opioid use – *CYP2B6*** | | | | | | |
| --- | --- | --- | --- | --- | --- | --- |
| **SNP** | **N** | **A1** | **TEST** | **OR** | **STAT** | **P** |
| **rs3745274** | 1129 | T | ADD | 0.82 | -1.61 | 0.11 |
|  |  |  | Age | 0.98 | -2.05 | 0.04 |
|  |  |  | Gender | 0.84 | -1.12 | 0.26 |
|  |  |  | Dose | 1.00 | -1.12 | 0.26 |
|  |  |  | Duration on MMT | 0.99 | -3.65 | 2.66E-4 |
| *Male* | 640 |  | ADD | 0.73 | -1.88 | 0.06** |
|  |  |  | Age | 0.98 | -2.15 | 0.03 |
|  |  |  | Dose | 1.00 | -1.79 | 0.07 |
|  |  |  | Duration on MMT | 0.99 | -2.60 | 9.29E-3 |
| *Female* | 489 |  | ADD | 0.95 | -0.25 | 0.80 |
|  |  |  | Age | 0.99 | -0.58 | 0.56 |
|  |  |  | Dose | 1.00 | 0.49 | 0.62 |
|  |  |  | Duration on MMT | 0.99 | -2.61 | 9.07E-3 |

| **Table C. Relapse – *OPRM1*** | | | | | | |
| --- | --- | --- | --- | --- | --- | --- |
| **SNP** | **N** | **A1** | **TEST** | **OR** | **STAT** | **P** |
| **rs73568641** | 944 | C | ADD | 0.98 | -0.20 | 0.84 |
|  |  |  | Age | 1.00 | 0.25 | 0.81 |
|  |  |  | Gender | 0.93 | -0.55 | 0.58 |
|  |  |  | Dose | 1.00 | 2.46 | 0.01 |
|  |  |  | Duration on MMT | 1.00 | 0.71 | 0.48 |
| *Male* | 530 |  | ADD | 0.96 | -0.21 | 0.84 |
|  |  |  | Age | 0.99 | -0.62 | 0.54 |
|  |  |  | Dose | 1.00 | 2.36 | 0.02 |
|  |  |  | Duration on MMT | 1.00 | -0.53 | 0.60 |
| *Female* | 414 |  | ADD | 1.04 | 0.17 | 0.86 |
|  |  |  | Age | 1.01 | 1.06 | 0.29 |
|  |  |  | Dose | 1.00 | 1.35 | 0.18 |
|  |  |  | Duration on MMT | 1.00 | 1.16 | 0.25 |
| **rs1799971** | 944 | G | ADD | 0.82 | -1.39 | 0.17 |
|  |  |  | Age | 1.00 | 0.29 | 0.77 |
|  |  |  | Gender | 0.92 | -0.62 | 0.54 |
|  |  |  | Dose | 1.00 | 2.49 | 0.01 |
|  |  |  | Duration on MMT | 1.00 | 0.72 | 0.48 |
| *Male* | 530 |  | ADD | 0.76 | -1.49 | 0.14 |
|  |  |  | Age | 1.00 | -0.54 | 0.59 |
|  |  |  | Dose | 1.01 | 2.44 | 0.01 |
|  |  |  | Duration on MMT | 1.00 | -0.53 | 0.59 |
| *Female* | 414 |  | ADD | 0.94 | -0.25 | 0.80 |
|  |  |  | Age | 1.01 | 1.05 | 0.29 |
|  |  |  | Dose | 1.00 | 1.32 | 0.19 |
|  |  |  | Duration on MMT | 1.00 | 1.16 | 0.25 |
| **rs10485058** | 944 | G | ADD | 1.10 | 0.66 | 0.51 |
|  |  |  | Age | 1.00 | 0.24 | 0.81 |
|  |  |  | Gender | 0.92 | -0.59 | 0.56 |
|  |  |  | Dose | 1.00 | 2.48 | 0.01 |
|  |  |  | Duration on MMT | 1.00 | 0.67 | 0.50 |
| *Male* | 530 |  | ADD | 1.02 | 0.11 | 0.91 |
|  |  |  | Age | 0.99 | -0.63 | 0.53 |
|  |  |  | Dose | 1.01 | 2.37 | 0.02 |
|  |  |  | Duration on MMT | 1.00 | -0.53 | 0.60 |
| *Female* | 414 |  | ADD | 1.15 | 0.68 | 0.50 |
|  |  |  | Age | 1.01 | 1.03 | 0.30 |
|  |  |  | Dose | 1.00 | 1.33 | 0.18 |
|  |  |  | Duration on MMT | 1.00 | 1.08 | 0.28 |

| **Table D. Relapse – *CYP2B6*** | | | | | | |
| --- | --- | --- | --- | --- | --- | --- |
| **SNP** | **N** | **A1** | **TEST** | **OR** | **STAT** | **P** |
| **rs3745274** | 944 | T | ADD | 0.91 | -0.81 | 0.42 |
|  |  |  | Age | 1.00 | 0.24 | 0.81 |
|  |  |  | Gender | 0.93 | -0.52 | 0.60 |
|  |  |  | Dose | 1.00 | 2.49 | 0.01 |
|  |  |  | Duration on MMT | 1.00 | 0.71 | 0.48 |
| *Male* | 530 |  | ADD | 0.86 | -1.00 | 0.32 |
|  |  |  | Age | 0.99 | -0.59 | 0.55 |
|  |  |  | Dose | 1.01 | 2.37 | 0.02 |
|  |  |  | Duration on MMT | 1.00 | -0.50 | 0.62 |
| *Female* | 414 |  | ADD | 1.07 | 0.37 | 0.71 |
|  |  |  | Age | 1.01 | 1.07 | 0.29 |
|  |  |  | Dose | 1.00 | 1.34 | 0.18 |
|  |  |  | Duration on MMT | 1.00 | 1.17 | 0.24 |

| **Table E. Methadone dose – *OPRM1*** | | | | | | |
| --- | --- | --- | --- | --- | --- | --- |
| **SNP** | **N** | **A1** | **TEST** | **BETA** | **STAT** | **P** |
| **rs73568641** | 1165 | C | ADD | -4.24 | -1.70 | 0.09** |
|  |  |  | Age | 0.41 | 3.26 | 1.16E-3 |
|  |  |  | Gender | -5.45 | -2.04 | 0.04 |
|  |  |  | Duration on MMT | 0.17 | 5.92 | 4.28E-09 |
|  |  |  | Weight | 1.29E-01 | 2.86 | 4.37E-3 |
| *Male* | 664 |  | ADD | -2.36 | -0.71 | 0.48 |
|  |  |  | Age | 0.489 | 2.93 | 3.49E-3 |
|  |  |  | Duration on MMT | 0.22 | 5.56 | 3.92E-08 |
|  |  |  | Weight | 0.13 | 1.53 | 0.13 |
| *Female* | 501 |  | ADD | -7.99 | -2.14 | 0.03* |
|  |  |  | Age | 0.28 | 1.46 | 0.14 |
|  |  |  | Duration on MMT | 0.12 | 2.80 | 5.36E-03 |
|  |  |  | Weight | 0.13 | 2.53 | 0.01 |
| **rs1799971** | 1165 | G | ADD | 0.20 | 0.07 | 0.94 |
|  |  |  | Age | 0.41 | 3.23 | 1.28E-3 |
|  |  |  | Gender | -5.28 | -1.98 | 0.05 |
|  |  |  | Duration on MMT | 1.74E-01 | 5.93 | 3.94E-09 |
|  |  |  | Weight | 0.13 | 2.88 | 4.00E-3 |
| *Male* | 664 |  | ADD | 2.59 | 0.69 | 0.49 |
|  |  |  | Age | 0.47 | 2.83 | 4.79E-3 |
|  |  |  | Duration on MMT | 0.22 | 5.57 | 3.81E-08 |
|  |  |  | Weight | 0.13 | 1.55 | 0.12 |
| *Female* | 501 |  | ADD | -4.92 | -1.06 | 0.29 |
|  |  |  | Age | 0.30 | 1.54 | 0.13 |
|  |  |  | Duration on MMT | 0.12 | 2.83 | 4.87E-03 |
|  |  |  | Weight | 0.13 | 2.55 | 0.01 |
| **rs10485058** | 1165 | G | ADD | -0.45 | -0.17 | 0.87 |
|  |  |  | Age | 0.41 | 3.24 | 1.24E-3 |
|  |  |  | Gender | -5.28 | -1.98 | 0.05 |
|  |  |  | Duration on MMT | 0.17 | 5.93 | 3.96E-09 |
|  |  |  | Weight | 0.13 | 2.87 | 4.18E-3 |
| *Male* | 664 |  | ADD | -0.50 | -0.14 | 0.89 |
|  |  |  | Age | 0.48 | 2.90 | 3.88E-3 |
|  |  |  | Duration on MMT | 0.22 | 5.56 | 3.93E-08 |
|  |  |  | Weight | 0.13 | 1.54 | 0.12 |
| *Female* | 501 |  | ADD | 0.24 | 0.06 | 0.95 |
|  |  |  | Age | 0.30 | 1.54 | 0.12 |
|  |  |  | Duration on MMT | 0.12 | 2.82 | 5.08E-03 |
|  |  |  | Weight | 0.13 | 2.53 | 0.01 |

| **Table F. Methadone dose – *CYP2B6*** | | | | | | |
| --- | --- | --- | --- | --- | --- | --- |
| **SNP** | **N** | **A1** | **TEST** | **BETA** | **STAT** | **P** |
| **rs3745274** | 1165 | T | ADD | 1.26 | 0.58 | 0.56 |
|  |  |  | Age | 0.41 | 3.24 | 1.23E-3 |
|  |  |  | Gender | -5.37 | -2.01 | 0.05 |
|  |  |  | Duration on MMT | 0.17 | 5.92 | 4.17E-09 |
|  |  |  | Weight | 1.30E-01 | 2.88 | 4.03E-3 |
| *Male* | 664 |  | ADD | -1.17 | -0.39 | 0.70 |
|  |  |  | Age | 0.49 | 2.91 | 3.74E-3 |
|  |  |  | Duration on MMT | 0.22 | 5.57 | 3.81E-08 |
|  |  |  | Weight | 0.14 | 1.59 | 0.11 |
| *Female* | 501 |  | ADD | 4.19 | 1.32 | 0.19 |
|  |  |  | Age | 0.31 | 1.61 | 0.11 |
|  |  |  | Duration on MMT | 0.12 | 2.82 | 5.00E-03 |
|  |  |  | Weight | 0.13 | 2.60 | 9.52E-3 |

**Between-Sex Association Tables**

| **Table G. *OPRM1* Gene** | | | | | | |
| --- | --- | --- | --- | --- | --- | --- |
| **Outcome** | **SNP** | **N** | **Minor Allele** | **OR/BETA** | **95% CI/SE** | **P** |
| **Continued opioid use** | **rs73568641** | 1129 | C | 1.48 | 0.62 3.51 | 0.37 |
|  | *SNPxSex* |  |  |  |  | 0.17 |
|  | **rs1799971** | 1129 | G | 1.44 | 0.53 3.95 | 0.48 |
|  | *SNPxSex* |  |  |  |  | 0.41 |
|  | **rs10485058** | 1129 | G | 0.82 | 0.32 2.12 | 0.69 |
|  | *SNPxSex* |  |  |  |  | 0.74 |
| **Relapse** | **rs73568641** | 944 | C | 0.92 | 0.39 0.43 | 0.83 |
|  | *SNPxSex* |  |  |  |  | 0.87 |
|  | **rs1799971** | 944 | G | 0.68 | 0.28 1.62 | 0.38 |
|  | *SNPxSex* |  |  |  |  | 0.66 |
|  | **rs10485058** | 944 | G | 0.93 | 0.40 2.18 | 0.87 |
|  | *SNPxSex* |  |  |  |  | 0.69 |
| **Methadone dose** | **rs73568641** | 1165 | C | 2.42 | 7.55 | 0.75 |
|  | *SNPxSex* |  |  |  |  | 0.35 |
|  | **rs1799971** | 1165 | G | 9.65 | 8.72 | 0.27 |
|  | *SNPxSex* |  |  |  |  | 0.25 |
|  | **rs10485058** | 1165 | G | -1.06 | 8.27 | 0.90 |
|  | *SNPxSex* |  |  |  |  | 0.94 |
| The minor alleles are also the reference and tested alleles. OR is odds ratio and BETA is the beta coefficient for the additive regression. 95% CI is the 95% confidence interval levels (lower, upper) and SE is the standard error. All results reported are odds ratios and 95% confidence intervals, except for the methadone dose outcomes, which are BETA coefficients and standard errors. P is the p-value for the t-statistic. The significance threshold is P<0.017.  *P<0.1  **P<0.05 | | | | | | |

| **Table H. *CYP2B6* Gene** | | | | | | |
| --- | --- | --- | --- | --- | --- | --- |
| **Outcome** | **SNP** | **N** | **Minor Allele** | **OR/BETA** | **95% CI/SE** | **P** |
| **Continued opioid use** | **rs73568641** | 1129 | C | 0.57 | 0.27 1.20 | 0.14 |
|  | *SNPxSex* |  |  |  |  | 0.31 |
| **Relapse** | **rs73568641** | 944 | C | 0.70 | 0.36 1.38 | 0.30 |
|  | *SNPxSex* |  |  |  |  | 0.42 |
| **Methadone dose** | **rs73568641** | 1165 | C | -6.90 | 6.68 | 0.30 |
|  | *SNPxSex* |  |  |  |  | 0.20 |
| The minor alleles are also the reference and tested alleles. OR is odds ratio and BETA is the beta coefficient for the additive regression. 95% CI is the 95% confidence interval levels (lower, upper) and SE is the standard error. All results reported are odds ratios and 95% confidence intervals, except for the methadone dose outcomes, which are BETA coefficients and standard errors. P is the p-value for the t-statistic. The significance threshold is P<0.017.  *P<0.1  **P<0.05 | | | | | | |
