# Supplementary material for: Implications of OPRM1 and CYP2B6 variants on treatment outcomes in methadone-maintained patients in Ontario: Exploring sex differences
Source: PLoS One. 2021 Dec 15;16(12):e0261201. doi: 10.1371/journal.pone.0261201 (PMC8673616; doi:10.1371/journal.pone.0261201)
Supplement: Attachment — Submitted filename: Response to Reviewers 2.docx [file pone.0261201.s004.docx]

Response to Reviewers

Title: Implications of OPRM1 and CYP2B6 variants on treatment outcomes in methadone-maintained patients in Ontario: Exploring sex differences

Date: November 25^th^, 2021

Journal Requirements:

Please review your reference list to ensure that it is complete and correct. If you have cited papers that have been retracted, please include the rationale for doing so in the manuscript text, or remove these references and replace them with relevant current references. Any changes to the reference list should be mentioned in the rebuttal letter that accompanies your revised manuscript. If you need to cite a retracted article, indicate the article’s retracted status in the References list and also include a citation and full reference for the retraction notice.

Response: The reference list has been reviewed and is complete with no cited papers that have been retracted to the knowledge of the authors. No changes to the reference list have been made.

**Comments to the Author**

1. If the authors have adequately addressed your comments raised in a previous round of review and you feel that this manuscript is now acceptable for publication, you may indicate that here to bypass the “Comments to the Author” section, enter your conflict of interest statement in the “Confidential to Editor” section, and submit your "Accept" recommendation.

Reviewer #1: All comments have been addressed

Reviewer #3: (No Response)

________________________________________

2. Is the manuscript technically sound, and do the data support the conclusions?

The manuscript must describe a technically sound piece of scientific research with data that supports the conclusions. Experiments must have been conducted rigorously, with appropriate controls, replication, and sample sizes. The conclusions must be drawn appropriately based on the data presented.

Reviewer #1: Partly

Reviewer #3: Yes

________________________________________

3. Has the statistical analysis been performed appropriately and rigorously?

Reviewer #1: Yes

Reviewer #3: Yes

________________________________________

4. Have the authors made all data underlying the findings in their manuscript fully available?

The PLOS Data policy requires authors to make all data underlying the findings described in their manuscript fully available without restriction, with rare exception (please refer to the Data Availability Statement in the manuscript PDF file). The data should be provided as part of the manuscript or its supporting information, or deposited to a public repository. For example, in addition to summary statistics, the data points behind means, medians and variance measures should be available. If there are restrictions on publicly sharing data—e.g. participant privacy or use of data from a third party—those must be specified.

Reviewer #1: Yes

Reviewer #3: Yes

________________________________________

5. Is the manuscript presented in an intelligible fashion and written in standard English?

PLOS ONE does not copyedit accepted manuscripts, so the language in submitted articles must be clear, correct, and unambiguous. Any typographical or grammatical errors should be corrected at revision, so please note any specific errors here.

Reviewer #1: Yes

Reviewer #3: Yes

________________________________________

6. Review Comments to the Author

Please use the space provided to explain your answers to the questions above. You may also include additional comments for the author, including concerns about dual publication, research ethics, or publication ethics. (Please upload your review as an attachment if it exceeds 20,000 characters)

Reviewer #1: (No Response)

Reviewer #3: Thank you for your response.

My comments have been addressed except for the addition of the range (min-max or interquartile) of methadone dose in Table 2. The number of significant digits (for methadone dose but not only) used in table 2 should also be reviewed.

And there is an inversion in table 2 in the Continued opioid use frequency.

Response: Thank you for your comments and review. In response to Reviewer #3’s comment, the range that was included in the previous revision as the difference between the highest and lowest methadone doses in Table 2 has been modified to show the “minimum-maximum” values. Table 2 values (of methadone dose and other descriptors) have been edited where applicable to show significant digits that are proportional to the magnitude of the SD values presented. Finally, the inversion in the “Continued opioid use” frequency in Table 2 has been corrected; thank you for noting that.

________________________________________

7. PLOS authors have the option to publish the peer review history of their article (what does this mean?). If published, this will include your full peer review and any attached files.

If you choose “no”, your identity will remain anonymous but your review may still be made public.

Do you want your identity to be public for this peer review? For information about this choice, including consent withdrawal, please see our Privacy Policy.

Reviewer #1: Yes: Wade Berrettini

Reviewer #3: No
